# Synthesis and characterization of nanocatalyst Cu^2+^/mesoporous carbon for amidation reactions of alcohols

**DOI:** 10.1038/s41598-023-36521-6

**Published:** 2023-06-22

**Authors:** Hossein Ghafuri, Peyman Hanifehnejad, Afsaneh Rashidizadeh, Zeinab Tajik, Hanieh Dogari

**Affiliations:** Catalysts and Organic Synthesis Research Laboratory, Department of Chemistry, Iran University of Science and Technology, Tehran, 16846‑13114 Iran

**Keywords:** Heterogeneous catalysis, Organic chemistry, Catalyst synthesis

## Abstract

In this research, mesoporous carbon (MC) with high efficiency (0.65 g yield from 1.0 g MCM-41 and 1.25 g sucrose) was successfully prepared by adding carbon precursor (sucrose) in a single step with ultrasonic waves, which reduces time and energy cost. Then, the Cu^2+^/Mesoporous carbon nanocatalyst (Cu^2+^/MC) was synthesized by adding Cu(NO_3_)_2_ in a single step and applied as a catalyst in amidation reactions of alcohols. Also, Cu^2+^/MC was characterized using different spectroscopic methods and techniques, including Fourier transform infrared spectroscopy (FT-IR), Field Emission Scanning Electron Microscopy (FE-SEM), N_2_ adsorption analysis (BET), X-ray diffraction analysis (XRD), Energy Dispersive X-ray (EDX), and Thermogravimetric Analysis (TGA). Moreover, to show the catalytic merits of Cu^2+^/MC, various primary and secondary amines and ammonium salts were applied in the amidation of alcohols. Easy synthesis method, recyclability, excellent yields (80–93%), and simple work-up are some noticeable strengths of using Cu^2+^/MC as a catalyst in this reaction.

## Introduction

Carbon-based materials have attracted much attention in recent decades due to their unique physicochemical, morphological, and structural properties. Also, they are utilized in various fields, including energy storage, drug delivery, sensing, photocatalysis, and imaging^1–3^. Among these materials, mesoporous carbons provide more active sites and supply a larger specific surface area due to their high porosity, pore structure, tailorable surface properties, and high chemical and thermal stability^4–7^. Mesoporous carbons can be synthesized by two methods, the soft-template method (organic-organic assembly arrangement) and the hard-template method (fills mesoporous silica template with a carbon precursor), which the hard-template method is more effective and straightforward. Mesoporous silicas are great candidates as solid templates and have high chemical and thermal stability. MCM-41, MCM-48, and SBA-15 are the common templates in the hard template method, which usually have two stages for adding carbon precursor to synthesize mesoporous carbons^8–10^.

Due to the high porosity of mesoporous carbons, they are applied in electrode materials, drug delivery, batteries, adsorbents, potassium storage, and catalyst supports^11–17^. Today, using heterogeneous catalysts is crucial in organic reactions because of the principles of green chemistry and recyclability. However, there are still challenges in some organic reactions, such as synthesizing amide bonds, including using toxic reagents, tedious work-up, and high reaction time^18–23^.

**Figure 1:**
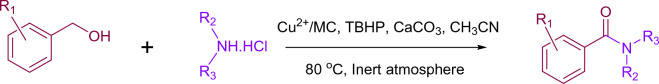
Cu^2+^/MC catalyzed tandem oxidative amidation of alcohols.

There are various amide compounds in nature and our bodies. The amide bond is the key functional group in many pharmaceutical and industrial products^24,25^. The most common methods to prepare amides are the reaction of acid chlorides, acid anhydrides, esters, and carboxylic acids with amines^26,27^. Release of an equivalent HCl, highly exothermic reactions, low yields of amide, and by-product formations are the problems of these reactions^28,29^. In this regard, developing different methods for synthesizing amide bonds is crucial in organic chemistry. In the last decade, scientists tried various methods to synthesize amide compounds, such as hydroamination of alkynes, aminocarbonylation, and amidation of aldehydes and carboxylic acids. Unfortunately, these methods have some defects, like utilizing expensive metals as catalysts, poor atom efficiency, high reaction time, low yields, and the creation of waste products that indicate the need for further research^30,31^. Lately, the amide bonds can be obtained by the amidation of alcohols, and by this method, the problems of synthesizing amide compounds by other methods have been removed^32–35^. In this research, based on the advantages of heterogeneous catalysts and the importance of amide compounds, we have synthesized Cu^2+^/MC and applied as a catalyst for the amidation of alcohols (Fig. 1).

## Experimental

### Materials

All materials were purchased from Merck and Sigma-Aldrich companies and applied without further purification. The ultrasonic bath was used by Becker vCLEAN company. The furnace from the Exciton Company was used to carbonize carbon precursors. FT-IR, ^13^C-NMR, and ^1^H-NMR spectra were taken by Shimadzu Fourier and Varian INOVA 500 MHz spectrum. The 9100 electrothermal devices were used for measuring melting points. The electron images were obtained by TE-SCAN Field Emission Scanning Electron Microscope. TESCAN VEGA//XMU, Philips PW1730, micromeritics ASAP 2020, STA6000, performed the EDX, XRD, BET, and TGA analyses.

### The procedure for the synthesis of MCM-41

**Figure 2:**
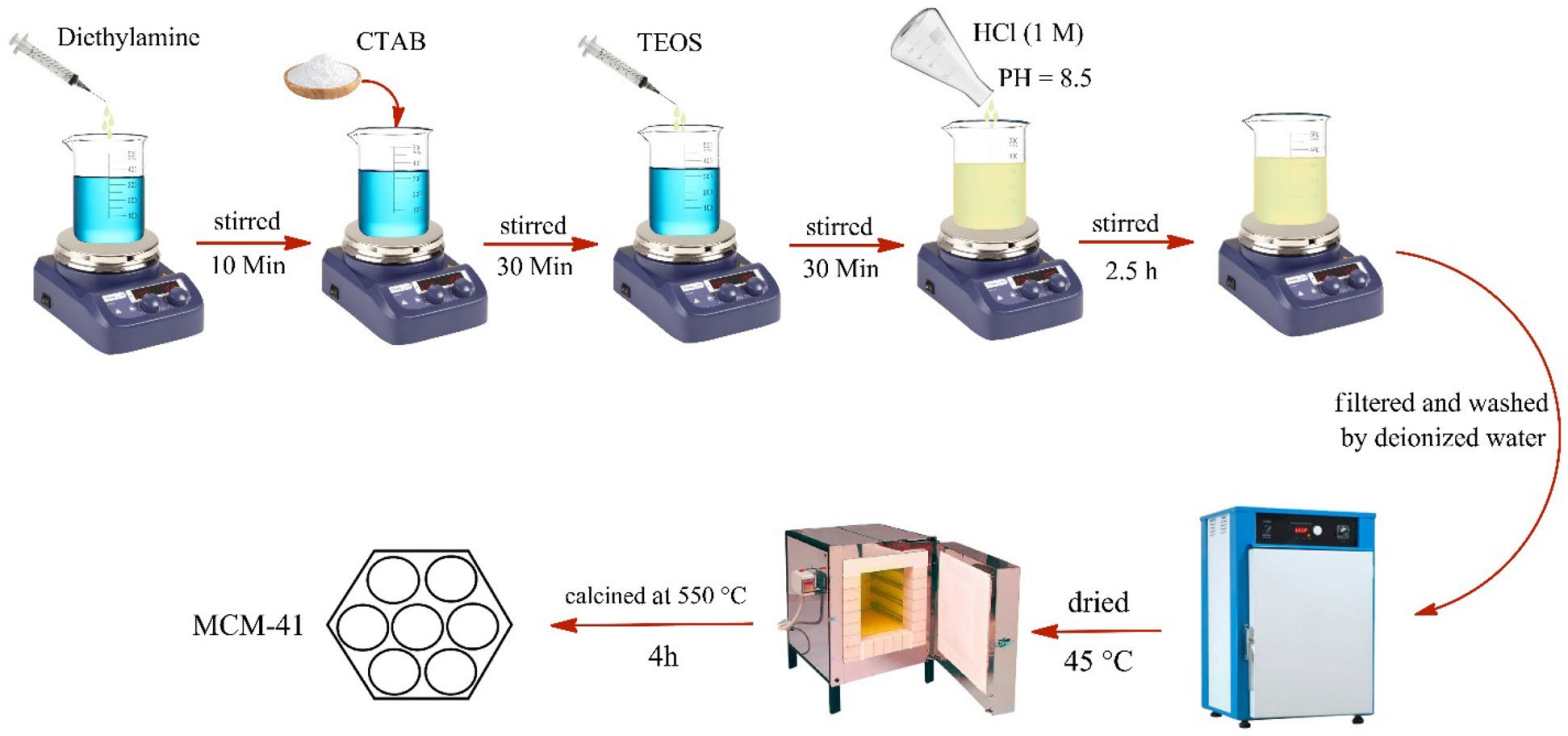
The graphical scheme for the synthesis of MCM-41.

The MCM-41 was prepared using the reported procedure^36^. First, diethylamine (4 mL) and deionized water (42 mL) were poured into a beaker and stirred for 10 min. Then, cetyltrimethyl ammonium bromide (CTAB, 1.47 g) was added to the mixture slowly. After 30 min, tetraethyl orthosilicate (TEOS, 4 mL) was added drop by drop, and the color of the mixture in this step changed to creamy. The pH of the mixture was set up to 8.5 by HCl (1 M) drop-wise and stirred for 2.5 h. After that, the mixture was filtered and washed with deionized water, dried at 45 $^\circ{\rm C}$ for 12 h, calcined at 550 °C for 4 h, and finally obtained the MCM-41 white powder. The graphical scheme of this procedure is shown in Fig. 2.

### The procedure for the synthesis of MC

**Figure 3:**
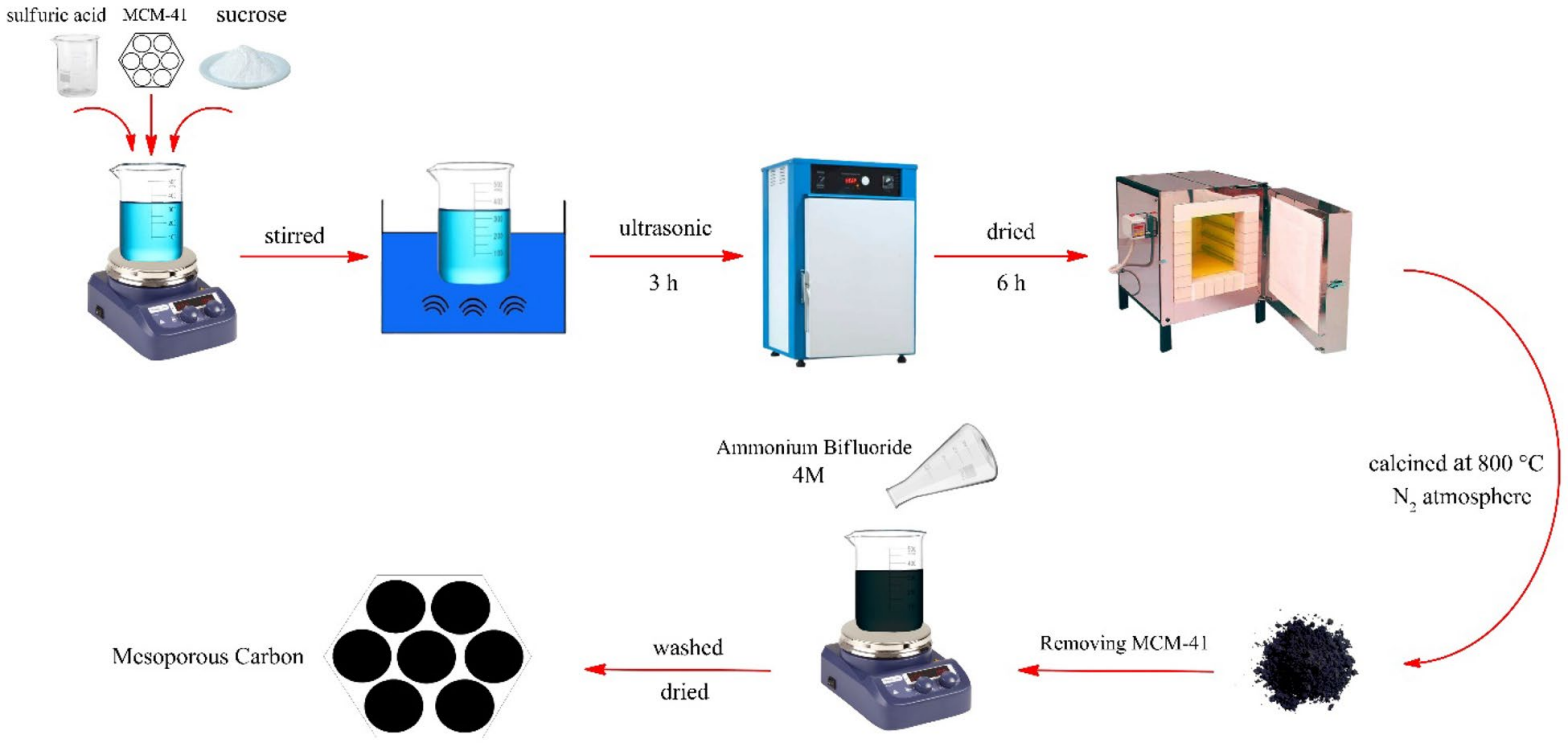
The graphical scheme for the synthesis of MC.

For this purpose, synthesized MCM-41 (1.0 g) was added slowly to a mixture of water (5.0 mL), sulfuric acid (1.5 mL), and sucrose (1.25 g) and stirred until a homogeneous solution has obtained. Afterward, the mixture was subjected to ultrasonic waves for 3 h until the sucrose precursor was completely inserted into the pores of MCM-41. Then, the mixture was placed in a 100 °C oven for 6 h to dry completely. At this stage, the color of the compound was changed to burnt brown or black. Next, the product was placed in the furnace under a nitrogen atmosphere at 800 °C by rate of 10 °C/min. The color of the obtained powder after the furnace changed to black. For removing the MCM-41, the obtained black powder was poured into a solution of ammonium bifluoride salt (40 mL, 4 M) and stirred. After 24 h, the black powder was separated by centrifugation, washed with water and ethanol, and dried in an 80 °C oven to obtain mesoporous carbon. The graphical scheme of this procedure is shown in Fig. 3.

### The procedure for the synthesis of Cu^2+^/MC

**Figure 4:**
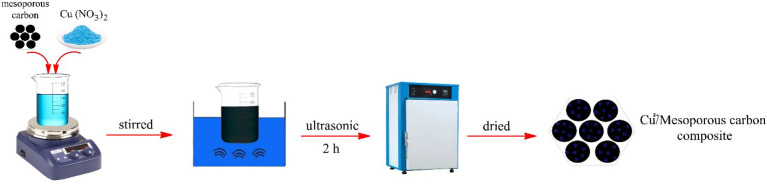
The graphical scheme for the synthesis of Cu^2+^/MC.

For synthesizing the Cu^2+^/MC composite, MC (1.0 g) was added to a solution of distilled water (10 mL) and Cu(NO_3_)_2_ (1.0 g) and stirred until the homogeneous mixture was obtained. The mixture is then subjected to ultrasonic waves for 2 h so that the coppers are placed into the holes of the MC. Afterward, the mixture was passed through the filter, washed with water and ethanol, and placed in an 80 °C oven to dry. The resulting black powder is a carbon composite of Cu^2+^/MC. The graphical scheme of this procedure is shown in Fig. 4.

### General procedure for direct amidation of benzyl alcohols

Benzyl alcohol (1.5 mmol), amine hydrochloride salt (1.0 mmol), CaCO_3_ (1.0 mmol), tert-butyl hydroproxide (TBHP, 4.0 Equiv), catalyst (20.0 mg), and acetonitrile (3 mL) as a solvent were added to a round bottom flask (25 mL) and refluxed for 4 h in an inert atmosphere. After completion of the reaction (monitored by TLC) catalyst was removed by filtration. After extracting the organic layer, the intended product has obtained by the anti-solvent method (ethyl acetate, n-hexane).

### Selected spectral data

#### *N*-benzyl-4-methoxybenzamide (3a)

FT-IR (KBr, cm^−1^): 3261, 1633, 1554, 1255, 1174 cm^−1^. ^1^H NMR (500 MHz, DMSO): **δ** H (ppm) = 3.80(3H, s, CH_3_), 4.45(2H, d, CH_2_), 7.01(2H, d, Ar–H), 7.31(5H, m, Ar–H), 7.89(2H, d, Ar–H), 8.89(1H, s, NH).

#### *N*-benzyl-*p*-chlorobenzamide (2a)

FT-IR (KBr, cm^−1^): 3290, 3060, 2918, 2806, 1639, 1548, 1257 cm^−1^. ^1^H NMR (500 MHz, DMSO): **δ** H (ppm) = 4.49(2H, d, NCH_2_), 7.24(2H, d, Ar–H), 7.32(5H, m, Ar–H), 7.31(5H, m, Ar–H), 7.92(2H, d, Ar–H), 9.15(1H, s, NH).

#### Benzamide (9a)

FTIR (KBr, cm^−1^): 3361, 3161, 1654, 1572, 1447, 1392, 1176, 629 cm^−1^. ^1^H NMR (500 MHz, DMSO): **δ** H (ppm) = 8.01(2H, s, NH_2_), 7.89(1H, m, Ar–H), 7.49(2H, m, Ar–H), 7.38(2H, m, Ar–H).

## Result and discussion

In this project, ultrasonic waves were used to add sucrose to the silica template in one step. The yield of MC for one-step synthesizing by reported procedures is about 0.35 g, while in this project, it is about 0.65 g.

**Figure 5:**
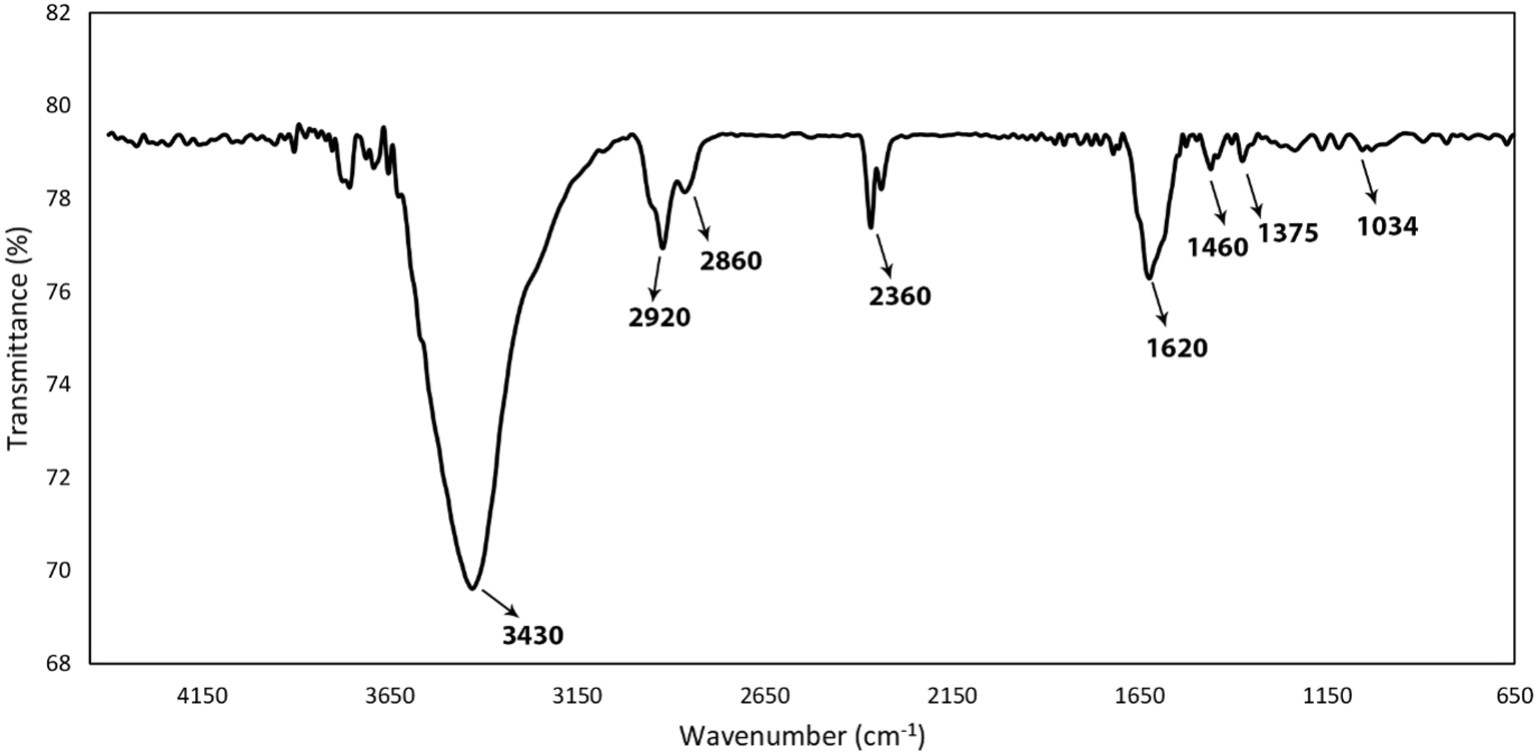
The FT-IR spectra of Cu^2+^/MC.

FT-IR spectroscopy was used to investigate the synthesis of Cu^2+^/MC and approve the presence of expected functional groups. The FT-IR spectrum of the Cu^2+^/MC is shown in Fig. 5. The peak around 3430 cm^−1^ could be described as the vibrational stretching of -OH groups of adsorbed H_2_O in the catalyst structure. The absorption peaks at about 1620 cm^−1^ are related to the tensile vibration of the C=C groups of carbon rings^37^. The peaks around 1460 cm^−1^ and 1375 cm^−1^ were characterized as bending vibrations of CH_3_ and CH_3_-CH_2_ groups, respectively. Also, there are the oop C–H bending vibrations around 1034 cm^−1^. The tensile vibration of the C–H groups corresponding to SP^3^ carbons appears at approximately 2860 cm^−1^ and 2920 cm^−1^. The peak at 2360 cm^−1^ is related to the absorption of CO_2_ by the device and not relevant to the Cu^2+^/MC^38,39^.

**Figure 6:**
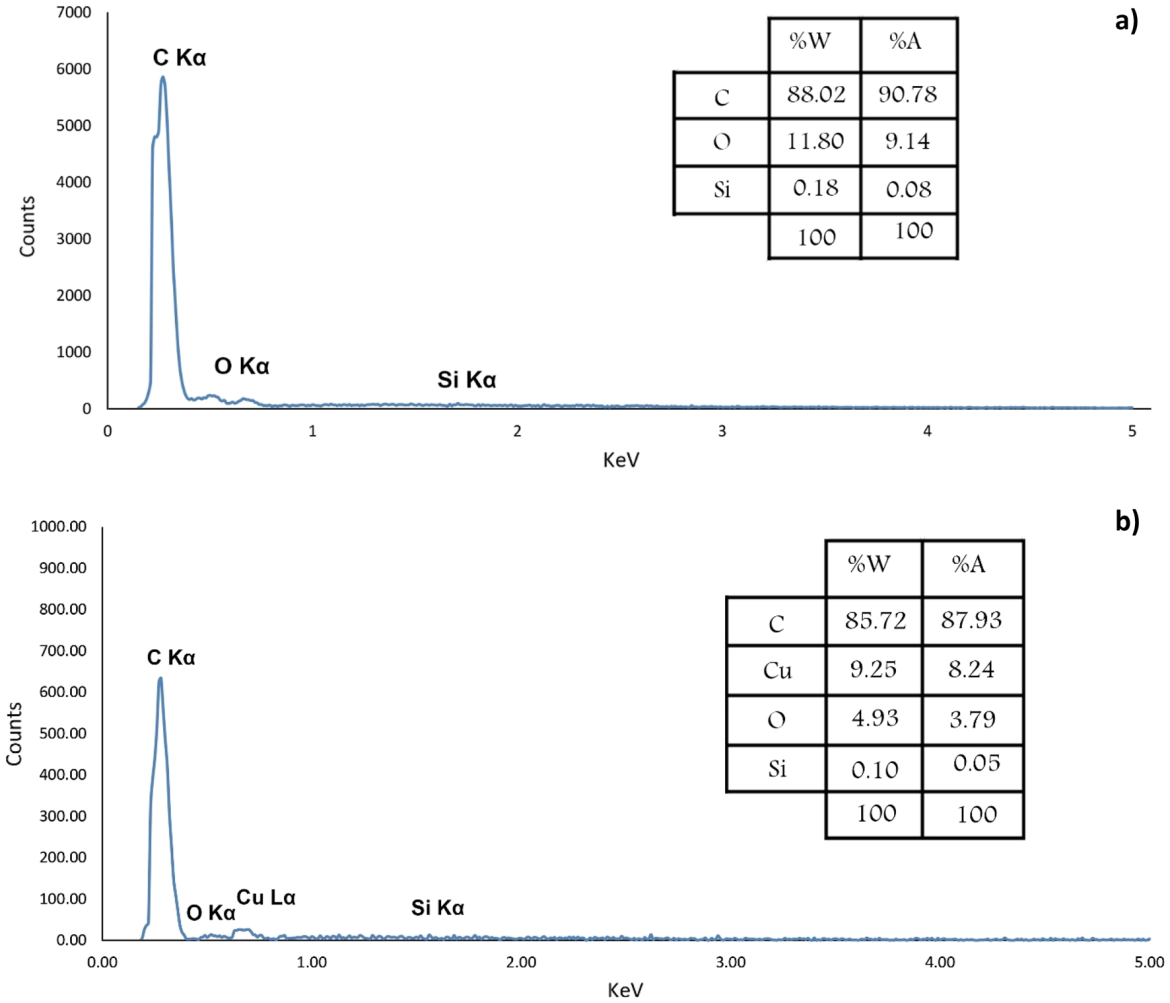
The EDX spectrum of **(a)** MC and **(b)** Cu^2+^/MC.

**Figure 7:**
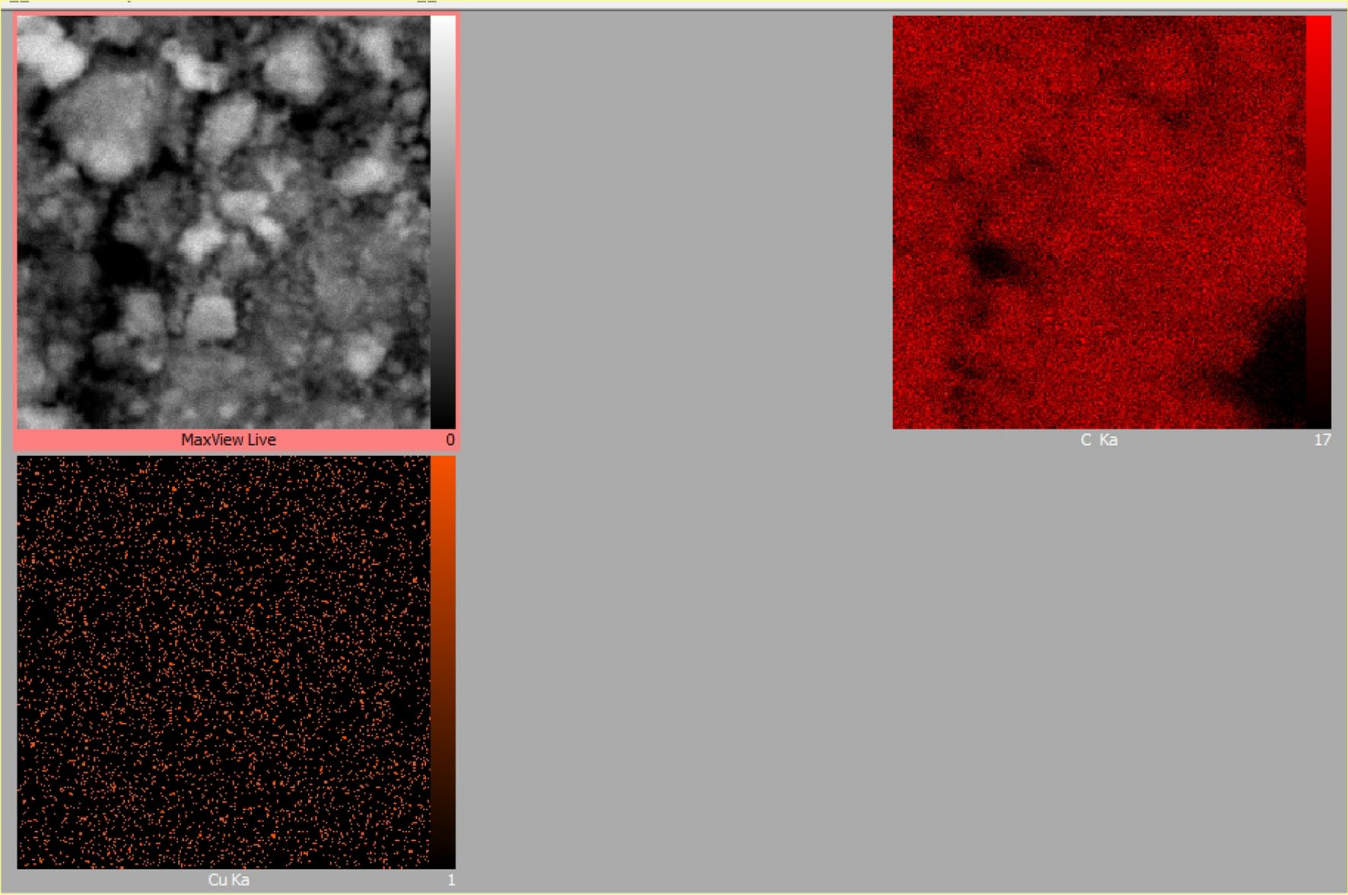
The elemental mapping of Cu^2+^/MC.

Energy Dispersive X-Ray analysis was performed to determine the elements in the MC and Cu^2+^/MC composition. As shown in Fig. 6, the presence of essential atoms, such as carbon, oxygen, and copper, in the fabricated MC and Cu^2+^/MC structure has also been verified. A small amount of silica is observed in the EDX analysis, which is because, after repeated washing, a small amount of silicon remains in the structure and is not completely removed^40,41^. A very high percentage of carbon indicates the successful synthesis of MC. Also, there is a low amount of oxygen in the structure assigned to the absorption of water at the surface of the MC pores. The Cu peak indicates loaded copper in pore channels of MC, where the catalyst's loaded Cu (II) is 3.5 wt% by ICP/OES analysis. Moreover, the elemental mapping of Cu^2+^/MC was taken to indicate the dispersity of the Cu (II) in the mesoporous carbon structure (Fig. 7).

**Figure 8:**
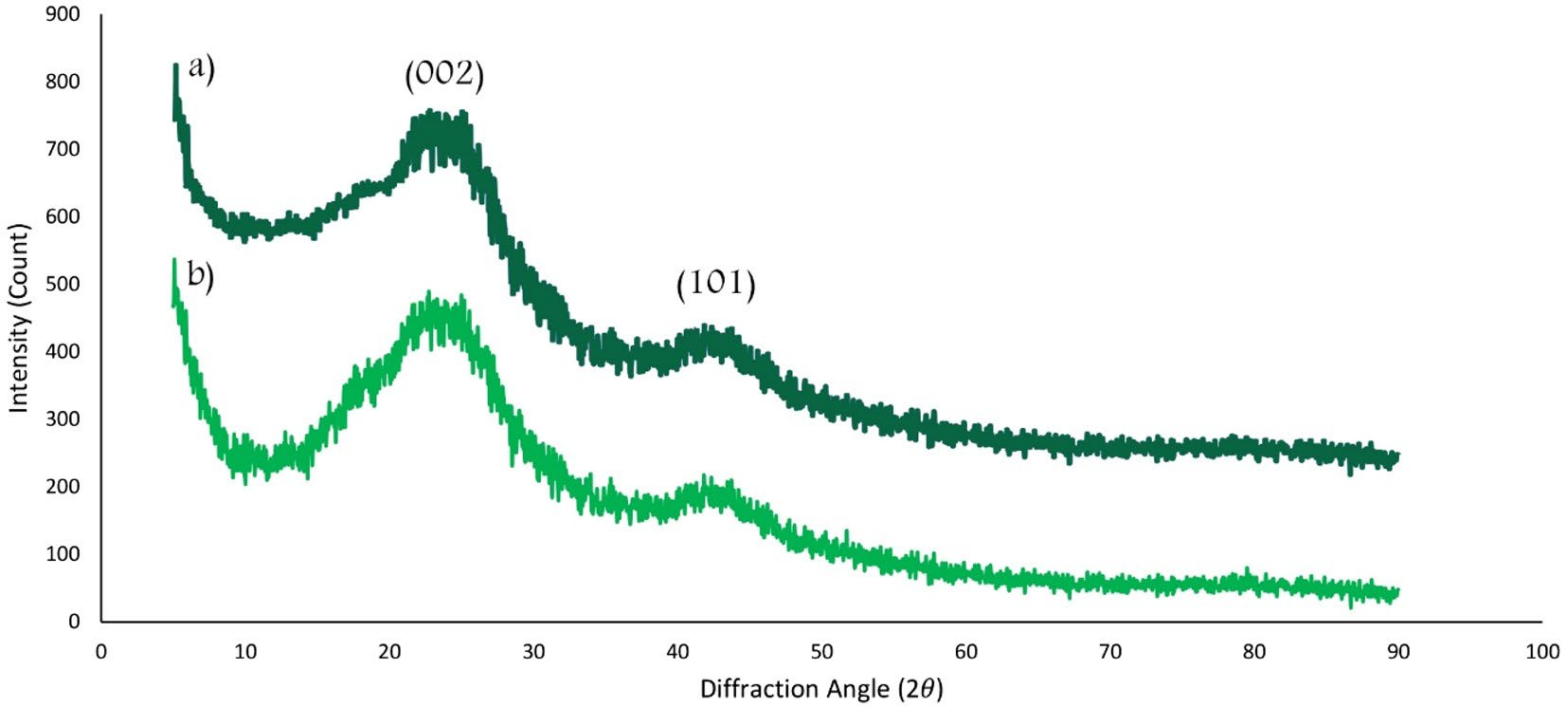
The XRD patterns of (**a**) MC and (**b**) Cu^2+^/MC.

The prepared MC and Cu^2+^/MC was crystallographically measured by wide-angle XRD Spectra in the range of 5^∘^ to 90^∘^ and shown in Fig. 8. It has two distinguish broad peaks at (002) and (101) (according to JCPDX index, No. 75-1621), which indicate the hexagonal graphitic structure of Cu^2+^/MC. There is no characteristic peak of Cu^2+^ in the XRD pattern. It may be due to high dispersion and its small size^42^. Also, the distinguish broad peaks of MC are retained after synthesizing Cu^2+^/MC.

**Figure 9:**
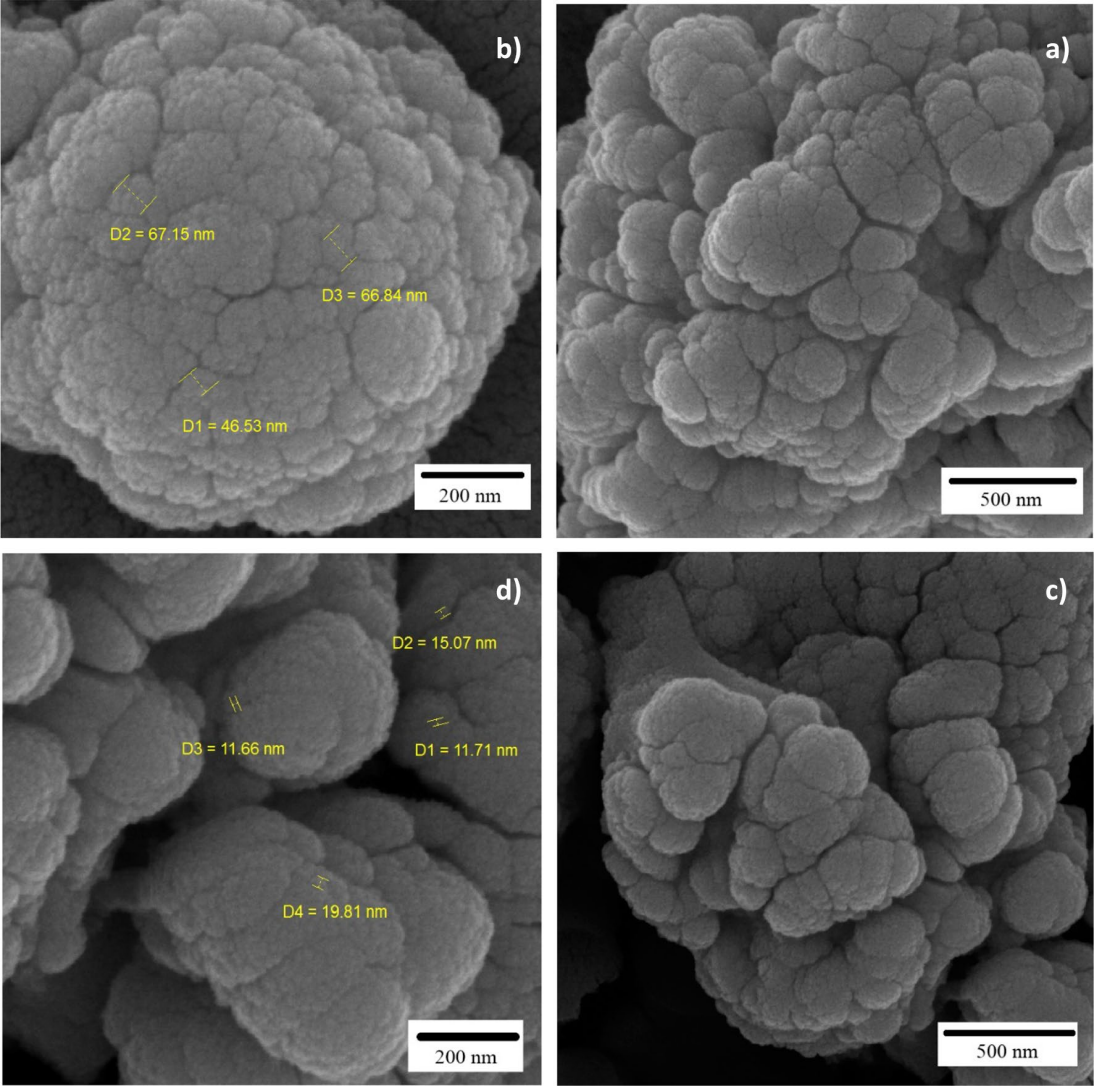
FE-SEM micrographs of (**a,b**) MC and (**c,d**) Cu^2+^/MC.

The morphology of MC (Fig. 9, a and b) and Cu^2+^/MC (Fig. 9c, d) were observed by Field Emission Scanning Electron Microscopy. FE-SEM Images were shown that MCM-41 acts as a template and forms mesoporous carbon particles on its surface. The morphology of these particles is interconnected like a network structure. Cu^2+^ species are uniformly distributed inside the structure of MC, and the structure is layered on top of each other, which XRD analysis is evidence of this claim.

**Figure 10:**
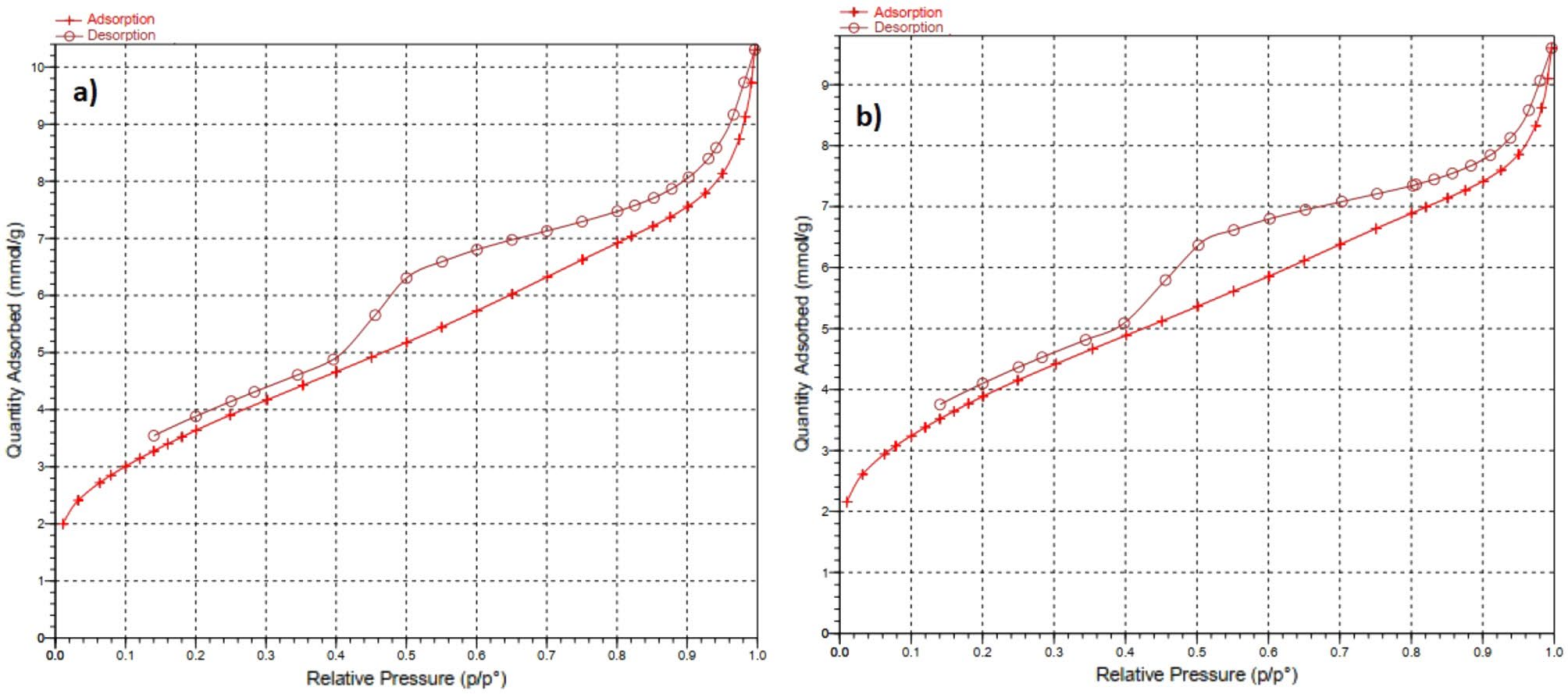
Isotherm linear plots of (**a**) MC and (**b**) Cu^2+^/MC.

To measure the surface area and pore size of the MC and Cu^2+^/MC, we used N_2_ adsorption analysis and showed the results in Fig. 10. Also, the Table 1 is shown the MC and Cu^2+^/MC surface area (300.0553, 318.8345 m^2^/g), pore size (40.3784, 36.2053 Å), and pore volume (0.302893, 0.288588 cm^3^/g). Figure 9a, b show the N_2_ adsorption and desorption isotherm diagrams of the MC and Cu^2+^/MC, respectively. These isotherms are similar to type (IV) isotherms that prove the mesoporous structure of synthesized compounds. According to Table 1, the pore size and the pore volume of Cu^2+^/MC have been reduced compared to MC, indicating copper trapping in the pores of mesoporous carbon. However, it can be observed that the surface area of Cu^2+^/MC has increased compared to MC due to the existence of copper on the crater of the pores.

**Table 1:** Surface area, pore size, and pore volume of MC and Cu^2+^/MC.

Entry	Name	S_(BET)_ (m^2^/g)	Pore size (Å)	Pore volume (cm^3^/g)
1	MC	300.0553	40.3784	0.302893
2	Cu^2+^/MC	318.8345	36.2053	0.288588

**Figure 11:**
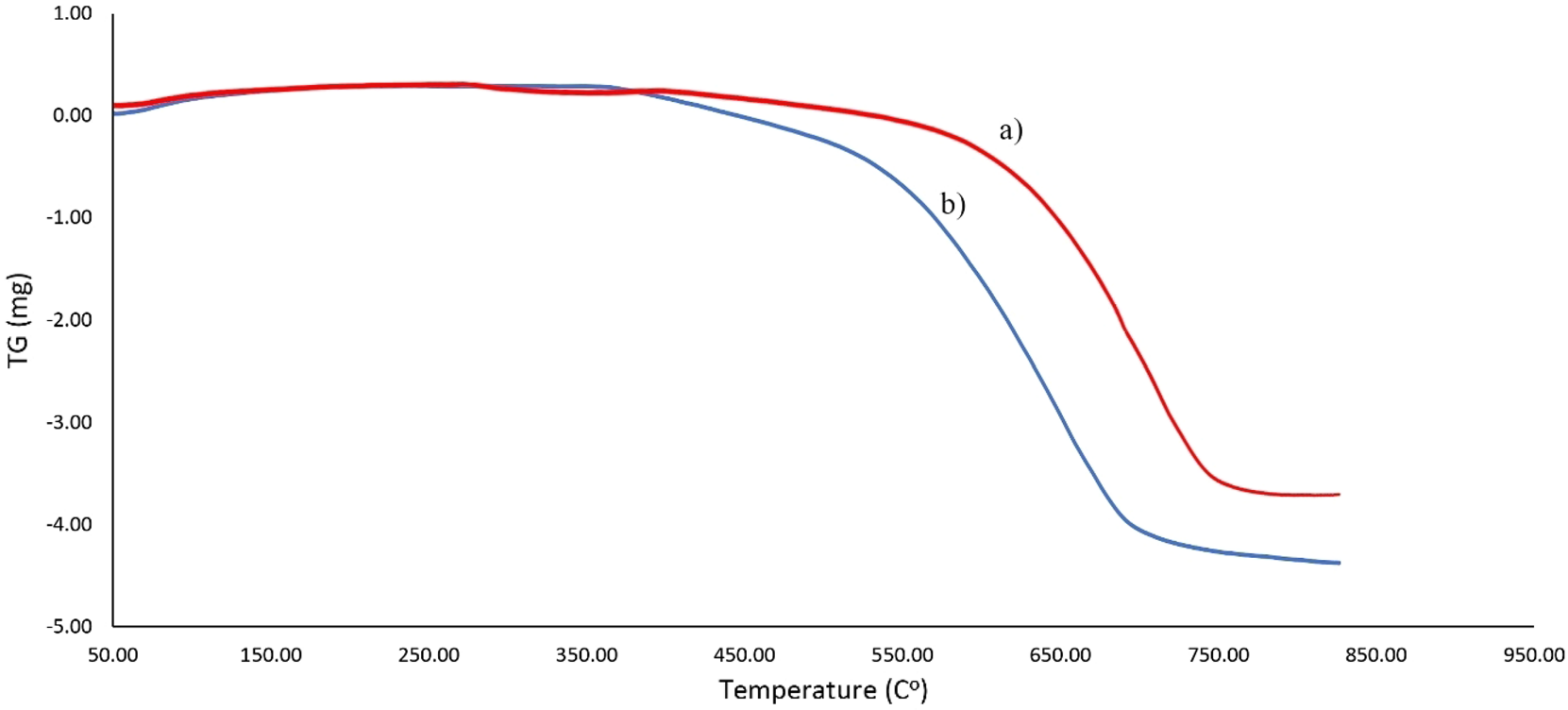
The TGA pattern of (**a**) MC and (**b**) Cu^2+^/MC.

Thermogravimetric analysis was used to investigate the thermal resistance of MC and Cu^2+^/MC, and the results were reported in Fig. 11a, b, respectively. According to Fig. 11a, the weight loss stage at an approximate temperature of 550 °C to 750 °C is due to the decomposition of the mesoporous carbon structure. The uniformity of the pattern before approximately 400 °C indicates the temperature resistance of Cu^2+^/MC. In addition, Cu^2+^/MC exhibits lower thermal stability than MC due to the distribution of Cu(II) in the MC structure. Also, there are no functional groups on the surface detected to degradation.

### Application of the catalyst in organic synthesis

For achieving the optimal conditions and the maximum efficiency of the Cu^2+^/MC catalyst, various parameters such as the amount of catalyst, different solvents, and the reaction temperature have been investigated and reported in Table 2. For catalyzing tandem oxidative amidation of benzyl alcohols, benzylamine hydrochloride (1.0 mmol) and benzyl alcohol (1.5 mmol) were considered as a model reaction, and the intended product yield was obtained by the anti-solvent method (ethyl acetate, n-hexane). First, this reaction was investigated without the catalyst and applied TBHP and H_2_O_2_ at 80 °C. According to the monitoring of TLC's reaction, no product was observed, which shows the catalyst's importance in this reaction (Table 2, entries 1 and 2). Then, by adding the catalyst (10 mg) to the reaction mixture, in the presence of solvent and without oxidant, it was observed that the desired product was not formed (Table 2, entry 3). It shows that adding the catalyst and oxidant both together would cause the reaction to proceed. In the following, in the presence of the catalyst, solvent, TBHP as an oxidant, and at room temperature, the reaction product's yield showed an efficiency of 23% (Table 1, entry 4). Increasing the reaction temperature from 25 to 80 °C improves the product's yield and leads to the reaction progressing and achieving higher efficiency. However, the 80 °C temperature is optimal, and above this temperature does not increase the yield and reduces product yields to 72% (Table 2, entries 5 and 6). The reason is due to the effect of this factor on the oxidation of benzyl alcohol to benzoic acid, which happens at higher temperatures. Adding the amount of catalyst from 10 to 20 mg increased the yield of the intended product (Table 2, entries 7 and 8). When the amount of catalyst increases to 30 mg at 80 °C, there is no change in the amide product (Table 2, entry 9). Various bases such as K_2_CO_3_, CaCO_3_, and Na_2_CO_3_ have also been used. Still, only CaCO_3_ leads to the progress of the reaction due to the slow formation of amine and the absence of the undesirable amine oxidation reaction (Table 2, entries 10 and 11). Subsequently, various oxidants besides TBHP, like H_2_O_2_ and O_2_, were exanimated to evaluate their effect on the reaction (Table 2, entries 12 and 13). Among the reported oxidants, tert-butyl hydroperoxide (TBHP) has shown a higher product yield for tandem oxidative amidation reaction. DMF and DMSO were also studied for this reaction as solvents. The best result was acetonitrile, and the other solvents did not give acceptable efficiencies (Table 2, entries 14 and 15).

**Table 2:** Optimizing the reaction conditions for the tandem oxidative amidation.

Entry	Solvent	Oxidant	Base	Amount of catalyst (mg)	Temperature (${^\circ{\rm C} }$)	Yield^a^ (%)
1	CH_3_CN	TBHP	CaCO_3_	–	80	–
2	CH_3_CN	H_2_O_2_	CaCO_3_	–	80	–
3	CH_3_CN	–	CaCO_3_	10	80	–
4	CH_3_CN	TBHP	CaCO_3_	10	25	23
5	CH_3_CN	TBHP	CaCO_3_	10	60	47
6	CH_3_CN	TBHP	CaCO_3_	10	80	72
**7**	CH_3_CN	TBHP	CaCO_3_	20	80	89
8	CH_3_CN	TBHP	CaCO_3_	20	100	73
9	CH_3_CN	TBHP	CaCO_3_	30	80	89
10	CH_3_CN	TBHP	K_2_CO_3_	20	80	–
11	CH_3_CN	TBHP	Na_2_CO_3_	20	80	–
12	CH_3_CN	H_2_O_2_	CaCO_3_	20	80	32
13	CH_3_CN	O_2_	CaCO_3_	20	80	27
14	DMF	TBHP	CaCO_3_	20	80	52
15	DMSO	TBHP	CaCO_3_	20	80	67

Reaction conditions: benyzlamine hydrochloride (1.0 mmol), benzyl alcohol(1.5 mmol), catalyst, solvent (3.0 mL), base (1 mmol), oxidant(70 wt% in H_2_O, 4 equiv), under N_2_ atmosphere at 80 °C for 4 h.

^a^The yields relate to the isolated product.

For evaluation of which component had the final effect on the reaction's catalyst, this reaction was performed by MC, Cu(NO_3_)_2_, and MC with copper salt, and the results are shown in Table 3. The results confirm that Cu^2+^/MC catalyzes the tandem oxidative amidation reaction.

**Table 3:** Efficiency of Cu^2+^/Mesoporous carbon with its components.

Entry	Catalyst	Yield (%)^a^
1	Mesoporous carbon	–
2	Cu(NO_3_)_2_	–
3	Mesoporous carbon, Cu(NO_3_)_2_	26
4	Cu^2+^/MC	89

Reaction conditions: benyzlamine hydrochloride (1.0 mmol), benzyl alcohol(1.5 mmol), catalyst (20 mg), solvent (3.0 mL), base (1 mmol), oxidant(70 wt % in H_2_O, 4 equiv), under N_2_ atmosphere at 80 °C for 4 h.

^a^The yields relate to the isolated product.

For evaluating the catalytic performance of Cu^2+^/MC nanocatalyst, several alcohols with electron-drawing and electron-donating groups with different types of amine hydrochloride salts were studied under optimized conditions in high to excellent yields (Table 4).

**Table 4:** The synthesis of amide derivatives in optimized condition using Cu^2+^/MC.


Entry	Alcohol	Amine salt	Product	Yield (%)^a^	Mp. (°C) (Ref.)
1a		A		89	107–109^43^
2a		A		93	161–163^44^
3a		A		91	101–103^45^
4a		A		83	132–134^45^
5a		A		85	140–142^46^
6a		A		82	156–158^47^
7a		C		88	Oil
8a		D		86	Oil
9a		B		90	125–127^48^
10a		B		88	170–172^49^
11a		B		85	162–164^50^
12a		B		82	158–160^50^
13a		B		89	198–200^50^
14a		B		80	189–191^49^

Reaction conditions: benyzlamine hydrochloride (1.0 mmol), benzyl alcohol(1.5 mmol), catalyst (20 mg), solvent (3.0 mL), base (1 mmol), oxidant(70 wt % in H_2_O, 4 equiv), under N_2_ atmosphere at 80 °C for 4 h.

^a^The yields relate to the isolated product.

Also, the Cu^2+^/MC was evaluated with some other catalysts to compare its catalytic performance, shown in Table 5. By comparing the efficiency of the final product, this catalyst has shown outstanding performance in this reaction.

**Table 5:** Comparing Cu^2+^/MC with other catalysts in amidation of alcohols.

Entry	Catalyst	Reaction condition	Time (h)	Yield (%)	Oxidant (eq)	Ref
1	FeCl_2_.4H_2_O	CH_3_CN, 80 °C	4	63	4	^33^
2	Ru(bpy)_3_Cl_2_	AcOEt, rt	72	79	1.2	^34^
3	NaI	CH_3_CN, 80 °C	4	87	8	^32^
4	Cu^2+^/MC	CH_3_CN, 80 °C	4	89	4	This work

## Mechanism

**Figure 12:**
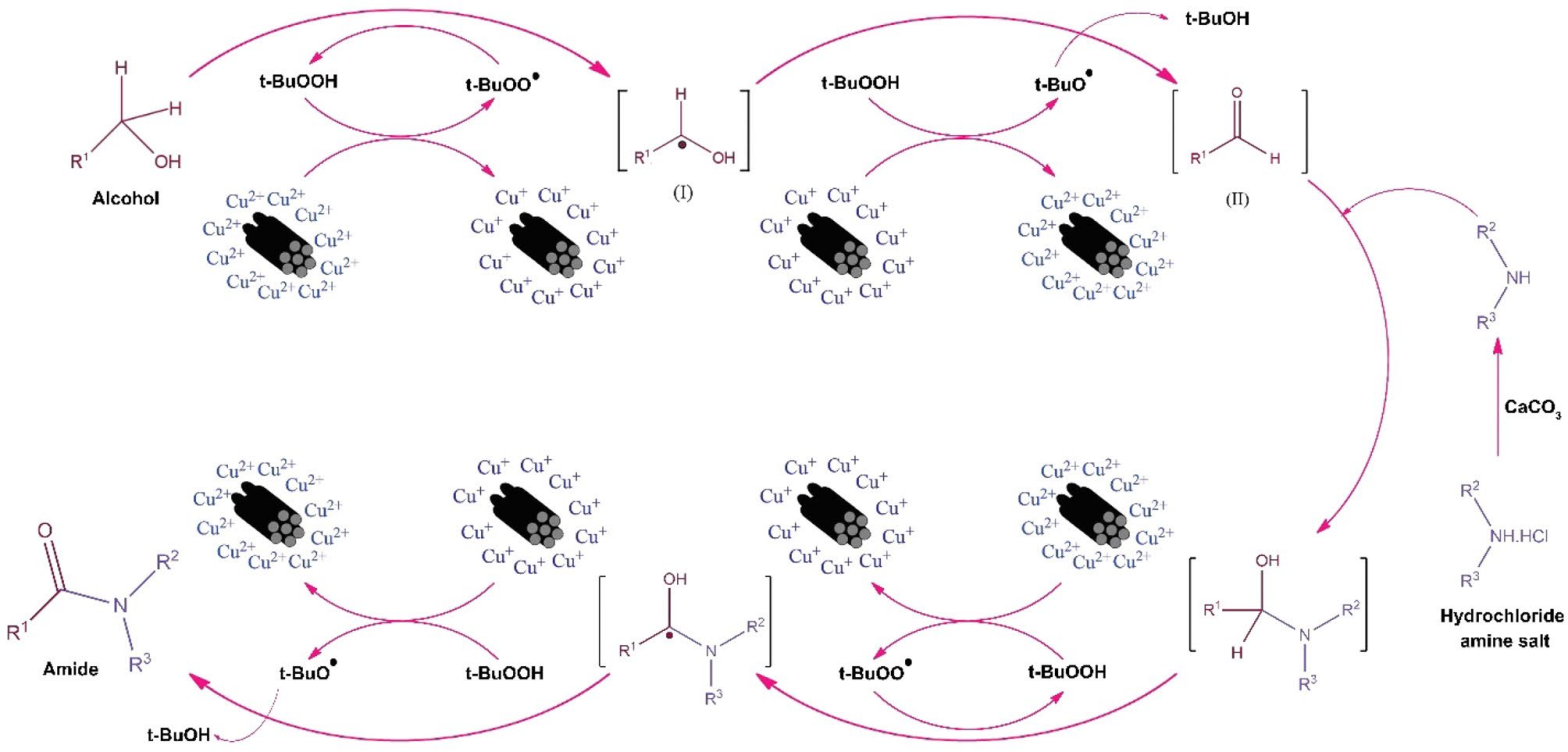
The proposed mechanism of oxidative amidation of alcohols.

In this reaction, it is considered that the oxidation of alcohol and the formation of aldehyde as the intermediate proceeds through a radical mechanism in the presence of Cu^2+^/MC and TBHP. The obtained aldehyde enters the reaction with the free amine in the environment obtained by the deprotonation of its salt by calcium carbonate, and the carbinolamine (III) intermediate is obtained. Afterward, intermediate (IV) would obtain by the reaction of radical TBHP and carbinolamine intermediate. Finally, this intermediate is oxidized, and the desired product is obtained through the radical mechanism. The overall scheme of this mechanism is given in Fig. 12. Also, the reaction was performed in an O_2_ atmosphere, and it was observed that no product was obtained. By approving this mechanism, Table 4 indicates that the withdrawing groups make the intermediate unstable, and the reaction yield would be increased. Also, the electron donating groups make the intermediate stable, and the yield would be decreased.

### Reusability

**Figure 13:**
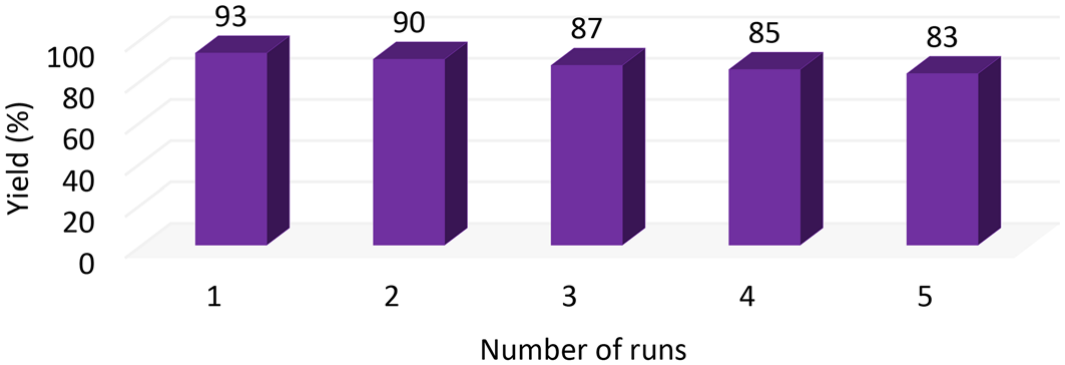
Recycling diagram of the Cu^2+^/MC.

**Figure 14:**
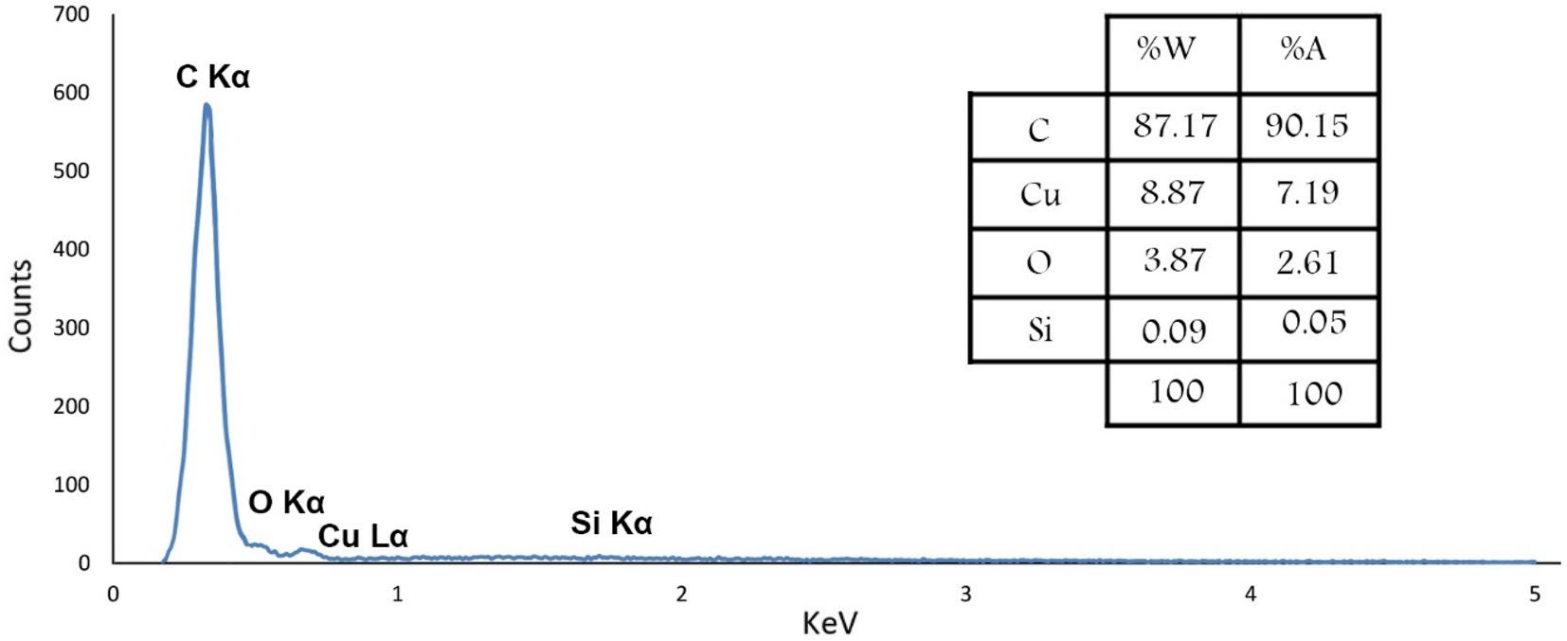
EDX analysis of recycled Cu^2+^/MC.

Reusability is one of the most critical factors of each catalytic system that highlights them as an efficient system due to the economic benefits and time-saving. For investigating catalyst recycling, the heterogeneous catalyst was first separated from the reaction mixture by filtration, washed with water and ethanol, and dried at 80 °C in an oven after each run to provide an opportunity for recycling experiments. It was observed that the catalyst could be reused at least five times with no significant reduction in its activity (Fig. 13). Also, EDX analysis was performed to determine the presence and stability of catalyst elements (Fig. 14). The result of the leaching of the Cu (II) after the recycling test was determined by ICP-OES analysis. The Cu (II) content in the synthesized catalyst was determined to be 3.5% before washing. This catalyst shows a content of 3.2% copper after reuse, confirming that the Cu (II) was not leached during the oxidative amidation reaction.

## Conclusion

To conclude, we have successfully synthesized Cu^2+^/MC composite with ultrasonication, reduced the carbonization steps, and increased the yield of MC. This catalyst's performance has shown promising results in the direct amidation of various electron-donating and electron-withdrawing groups of alcohols and different benzylic and primary amine salts. The oxidant in this reaction was TBHP which is non-toxic and decomposes to water and tert-butanol. High yield, short reaction time, mild conditions, easy separation, and catalyst recyclability are the advantages of using Cu^2+^/MC composite as a catalyst in the direct amidation of alcohols reaction (Supplementary Information).

## Supplementary Information

Supplementary Information.

## Data Availability

The datasets used and/or analysed during the current study available from the corresponding author on reasonable request.
