# Supplementary material for: Synthesis and characterization of nanocatalyst Cu2+/mesoporous carbon for amidation reactions of alcohols
Source: Sci Rep. 2023 Jun 22;13:10133. doi: 10.1038/s41598-023-36521-6 (PMC10287763; doi:10.1038/s41598-023-36521-6)
Supplement: Supplementary file 1 — Supplementary Information. [file 41598_2023_36521_MOESM1_ESM.docx]

**Synthesis and characterization of nanocatalyst Cu^2+^/** **Mesoporous carbon for amidation reactions of alcohols**

**Hossein Ghafuri^*^, Peyman Hanifehnejad, Afsaneh Rashidizadeh, Zeinab Tajik, Hanieh Dogari**

Catalysts and Organic Synthesis Research Laboratory, Department of Chemistry, Iran University of Science and Technology, Tehran 16846‑13114, Iran

* E-mail: ghafuri@iust.ac.ir

**SUPPLEMENTARY INFORMATION**

| **Page** | **Content** |
| --- | --- |
| 1 | Title page |
| 2 | Experimental |
| 7 | ICP Analysis |
| 8 | Selected spectral data |

**Figure 1.** Cu^2+^/MC catalyzed tandem oxidative amidation of alcohols

**Experimental**

**The procedure for the synthesis of MCM-41**

The MCM-41 was prepared using the reported procedure^36^. First, diethylamine (4 mL) and deionized water (42 mL) were poured into a beaker and stirred for 10 min. Then, cetyltrimethyl ammonium bromide (CTAB, 1.47 g) was added to the mixture slowly. After 30 min, tetraethyl orthosilicate (TEOS, 4 mL) was added drop by drop, and the color of the mixture in this step changed to creamy. The pH of the mixture was set up to 8.5 by HCl (1 M) drop-wise and stirred for 2.5 h. After that, the mixture was filtered and washed with deionized water, dried at 45 $℃$ for 12 h, calcined at 550 $℃$ for 4 h, and finally obtained the MCM-41 white powder. The graphical scheme of this procedure is shown in Fig. 2.


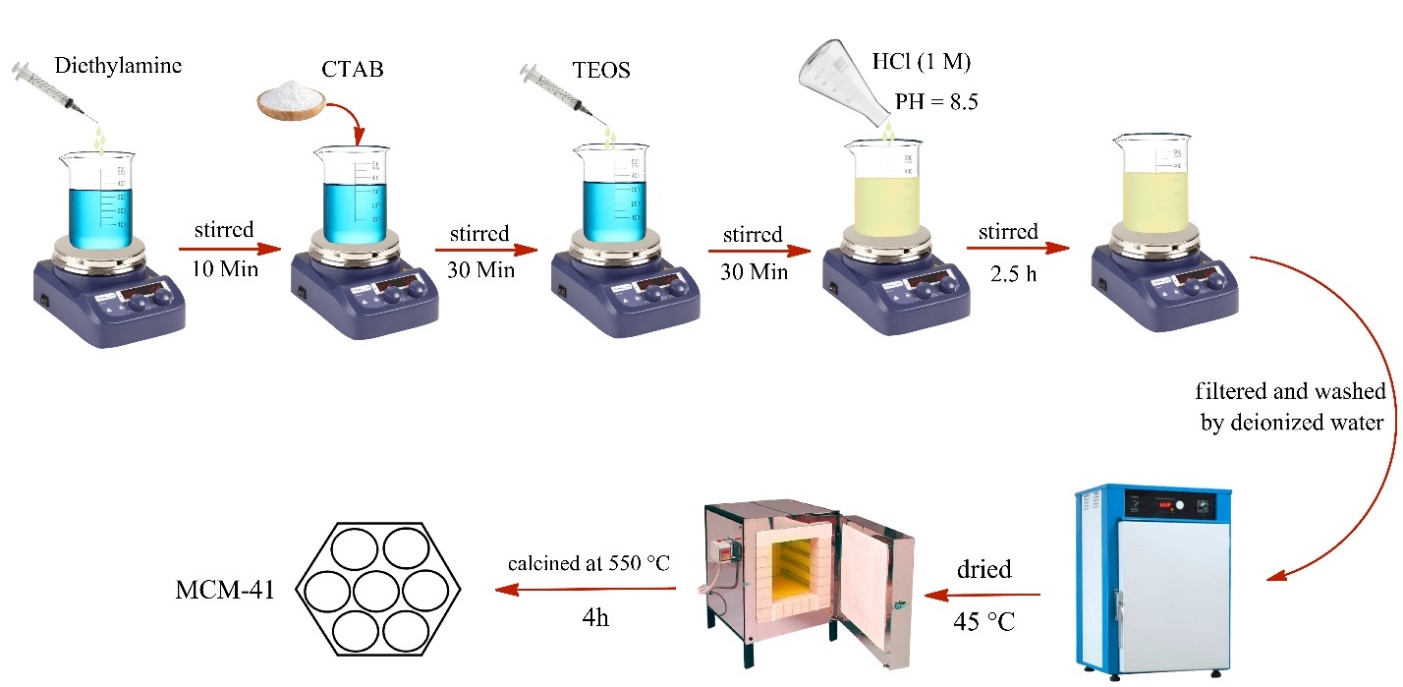


**Figure 2.** The graphical scheme for the Synthesis of MCM-41

**The procedure for the synthesis of MC**

For this purpose, synthesized MCM-41 (1.0 g) was added slowly to a mixture of water (5.0 mL), sulfuric acid (1.5 mL), and sucrose (1.25 g) and stirred until a homogeneous solution has obtained. Afterward, the mixture was subjected to ultrasonic waves for 3 h until the sucrose precursor was completely inserted into the pores of MCM-41. Then, the mixture was placed in a 100 $℃$ oven for 6 hours to dry completely. At this stage, the color of the compound was changed to burnt brown or black. Next, the product was placed in the furnace under a nitrogen atmosphere at 800 $℃$ by rate of 10 $℃$ per minute. The color of the obtained powder after the furnace changed to black. For removing the MCM-41, the obtained black powder was poured into a solution of ammonium bifluoride salt (40 mL, 4 M) and stirred. After 24 h, the black powder was separated by centrifugation, washed with water and ethanol, and dried in an 80 $℃$oven to obtain mesoporous carbon. The graphical scheme of this procedure is shown in Fig. 3.


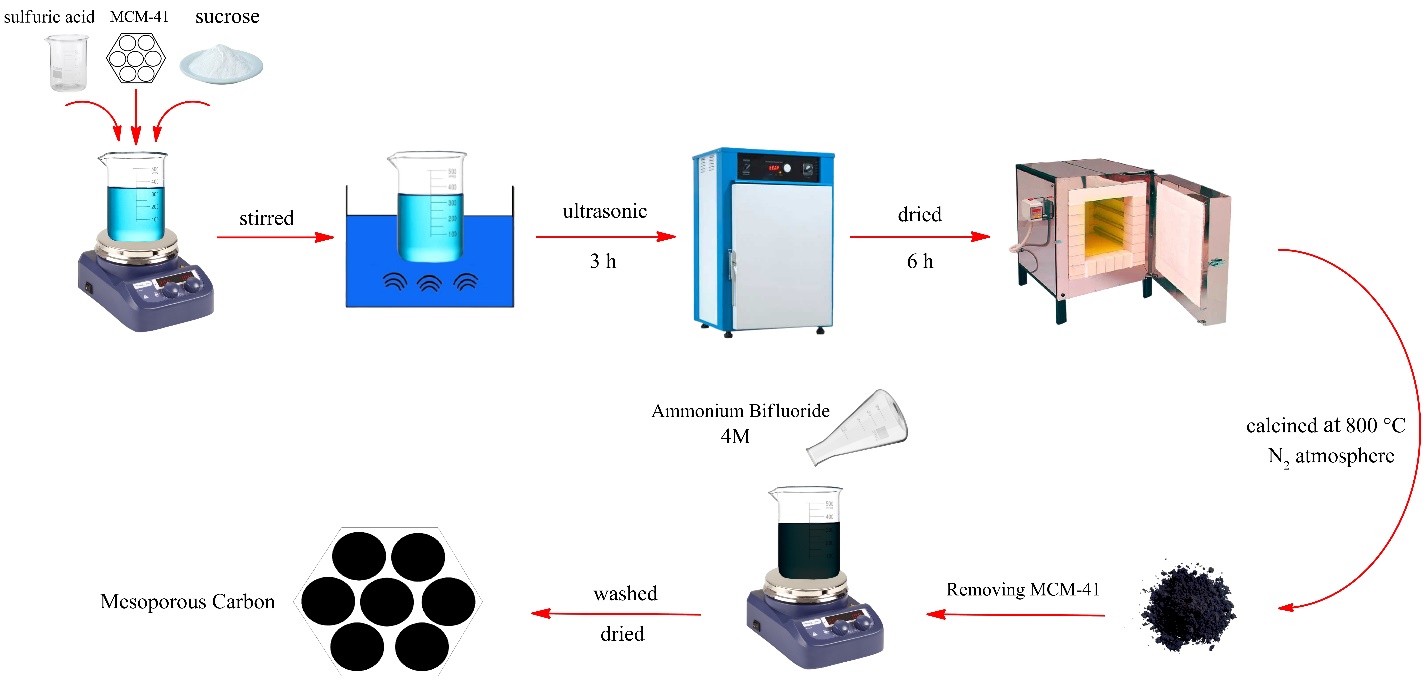


**Figure 3.** The graphical scheme for the Synthesis of MC

**The procedure for the synthesis of Cu^2+^/MC**

For synthesizing the Cu^2+^/MC composite, MC (1.0 g) was added to a solution of distilled water (10 mL) and Cu(NO_3_)_2_ (1.0 g) and stirred until the homogeneous mixture was obtained. The mixture is then subjected to ultrasonic waves for 2 h so that the coppers are placed in the holes of the MC. Afterward, the mixture was passed through the filter, washed with water and ethanol, and placed in an 80 $℃$ oven to dry. The resulting black powder is a carbon composite of Cu^2+^/MC. The graphical scheme of this procedure is shown in Fig. 4.


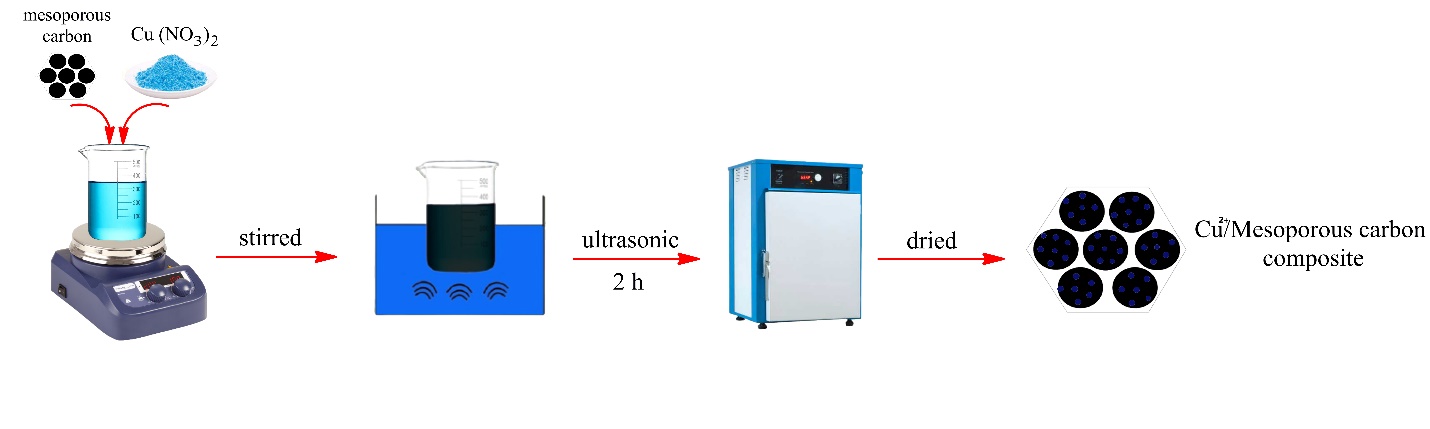


**Figure 4.** The graphical scheme for the Synthesis of Cu^2+^/MC

**General procedure for direct amidation of benzyl alcohols**

Benzyl alcohol (1.5 mmol), amine hydrochloride salt (1.0 mmol), CaCO_3_ (1.0 mmol), tert-butyl hydroproxide (TBHP, 4.0 Equiv), catalyst (20.0 mg), and acetonitrile (3 mL) as a solvent were added to a round bottom flask (25 mL) and refluxed for 4h in an inert atmosphere. After completion of the reaction (monitored by TLC) catalyst was removed by filtration. After extracting the organic layer, the intended product has obtained by the anti-solvent method (ethyl acetate, n-hexane).

**Table 1.** The synthesis of amide derivatives in optimized condition using Cu^2+^/MC^a^.

D

C

B

A

| Entry | Alcohol | Amine salt | Product | Yield (%)^b^ | Mp. (ºC)  [Ref.] |
| --- | --- | --- | --- | --- | --- |
| 1a |  | A |  | 89 | 107-109^43^ |
| 2a |  | A |  | 93 | 161-163^44^ |
| 3a |  | A |  | 91 | 101-103^45^ |
| 4a |  | A |  | 83 | 132-134^45^ |
| 5a |  | A |  | 85 | 140-142^46^ |
| 6a |  | A |  | 82 | 156-158^47^ |
| 7a |  | C |  | 88 | Oil |
| 8a |  | D |  | 86 | Oil |
| 9a |  | B |  | 90 | 125-127^48^ |
| 10a |  | B |  | 88 | 170-172^49^ |
| 11a |  | B |  | 85 | 162-164^50^ |
| 12a |  | B |  | 82 | 158-160^50^ |
| 13a |  | B |  | 89 | 198-200^50^ |
| 14a |  | B |  | 80 | 189-191^49^ |

^a^ Reaction conditions: benyzlamine hydrochloride (1.0 mmol), benzyl alcohol(1.5 mmol), catalyst (20 mg), solvent (3.0 mL), base (1 mmol), oxidant(70 wt % in H_2_O, 4 equiv), under N_2_ atmosphere at 80 $℃$ for 4 h.

^b^ The yields relate to the isolated product.

**ICP Analysis**

ICP-EOS analysis is use to find how much of a certain element exist in a sample. This analysis reports the amount of the element in ppm, which its Weight percent can be obtained by using the following formula:

$$WT\%= \frac{ppm}{10000}$$

Here, the Cu (II) content of the synthesized nanocomposite and recycled nanocomposite was calculated using ICP-EOS analysis, which includes 35000 and 32000 ppm, respectively. Using the mentioned formula, the weight percent of this element is as follows:

$$WT\%= \frac{35000}{10000}=3.5 \%$$

$$WT\%= \frac{32000}{10000}=3.2 \%$$

**Selected Spectral Data**

**N-benzyl-4-methoxybenzamide (3a)**

FTIR (KBr, cm^-1^): 3261, 1633, 1554, 1255, 1174 cm^-1^. ^1^H NMR (500 MHz, DMSO): δ H (ppm)= 3.80(3 H, s, CH_3_), 4.45(2H, d, CH_2_), 7.01(2H, d, Ar-H), 7.31(5H, m, Ar-H), 7.89(2H, d, Ar-H), 8.89(1H, s, NH)


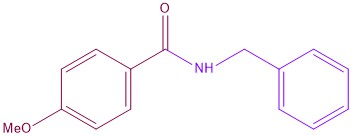

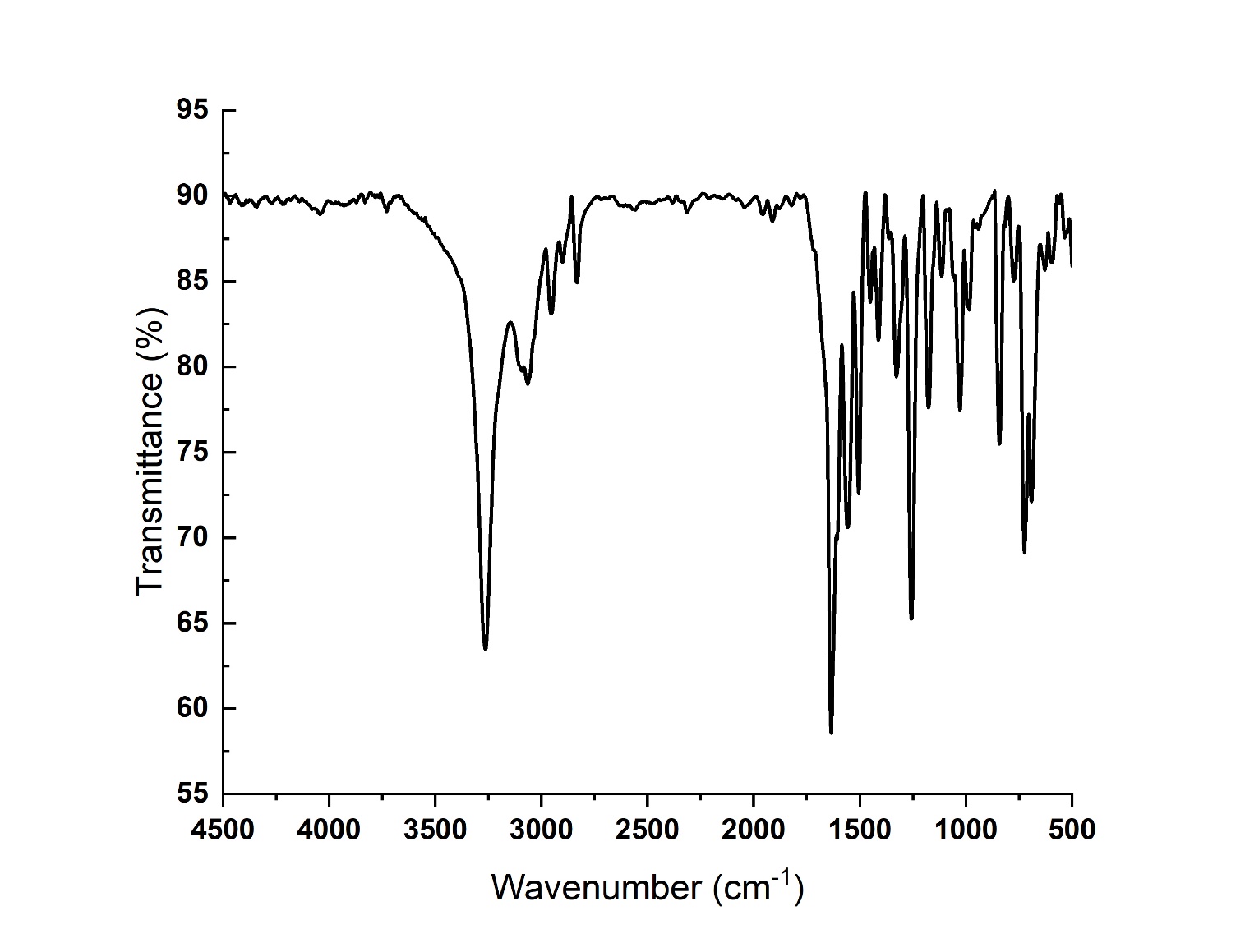


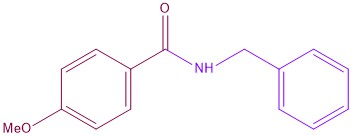

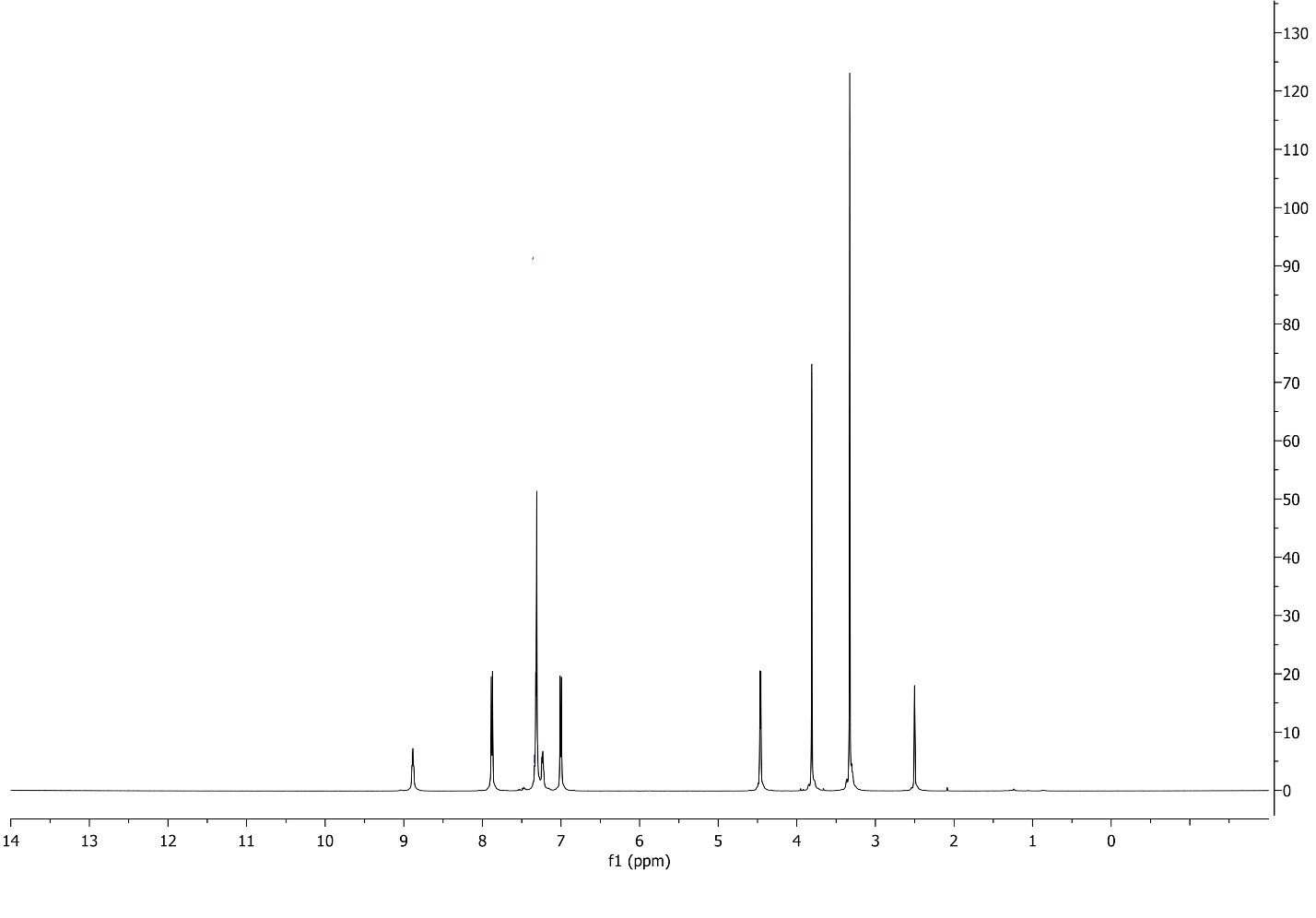


[**N-benzyl-p-chlorobenzamide**](javascript:) **(2a)**

FTIR (KBr, cm^-1^): 3290, 3060, 2918, 2806, 1639, 1548, 1257 cm^-1^. ^1^H NMR (500 MHz, DMSO): δ H (ppm)= 4.49(2H, d, NCH_2_), 7.24(2H, d, Ar-H), 7.32(5H, m, Ar-H), 7.31(5H, m, Ar-H), 7.92(2H, d, Ar-H), 9.15(1H, s, NH)


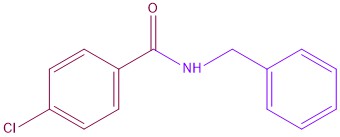

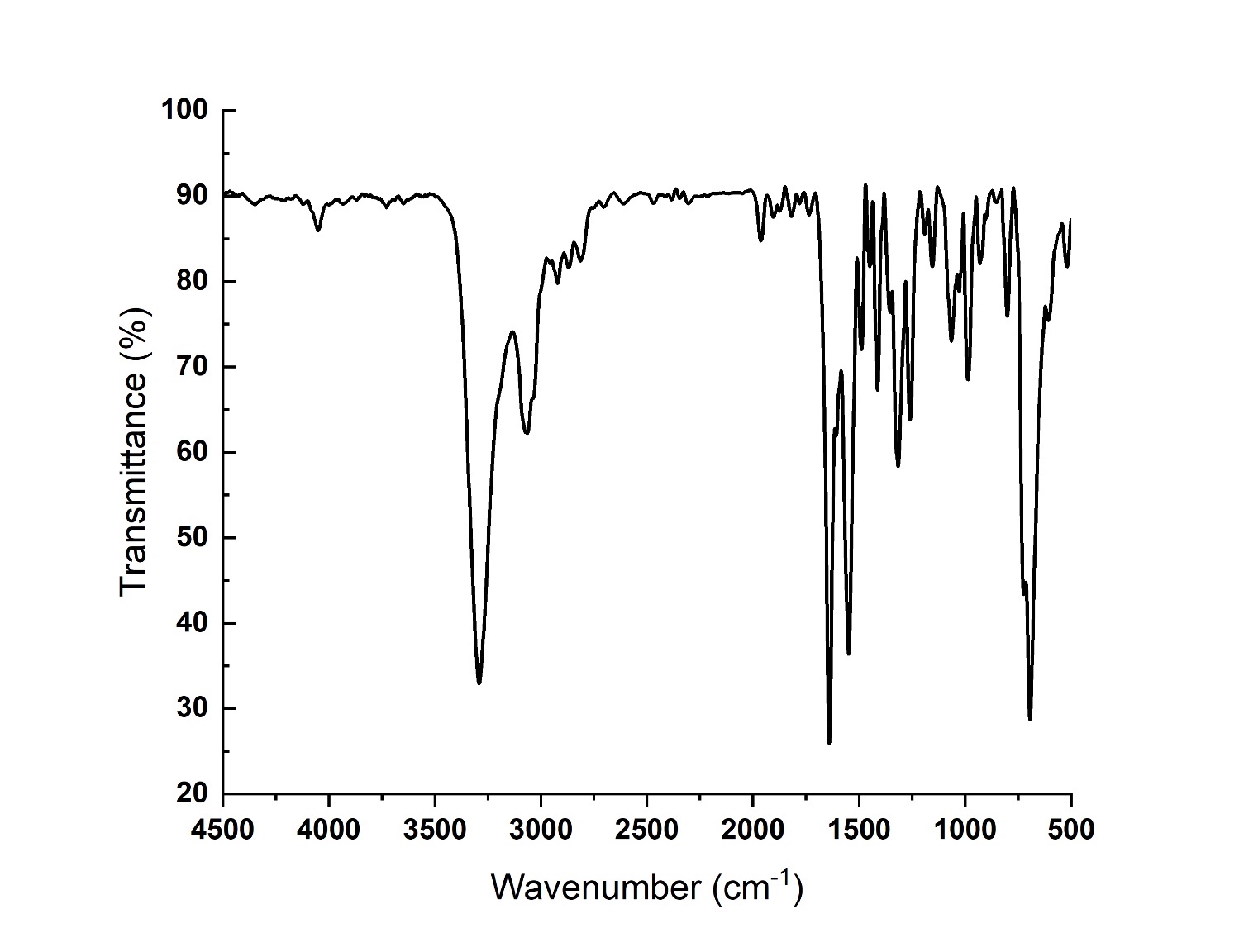


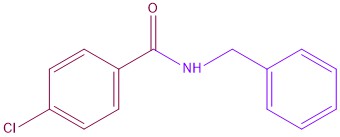
**
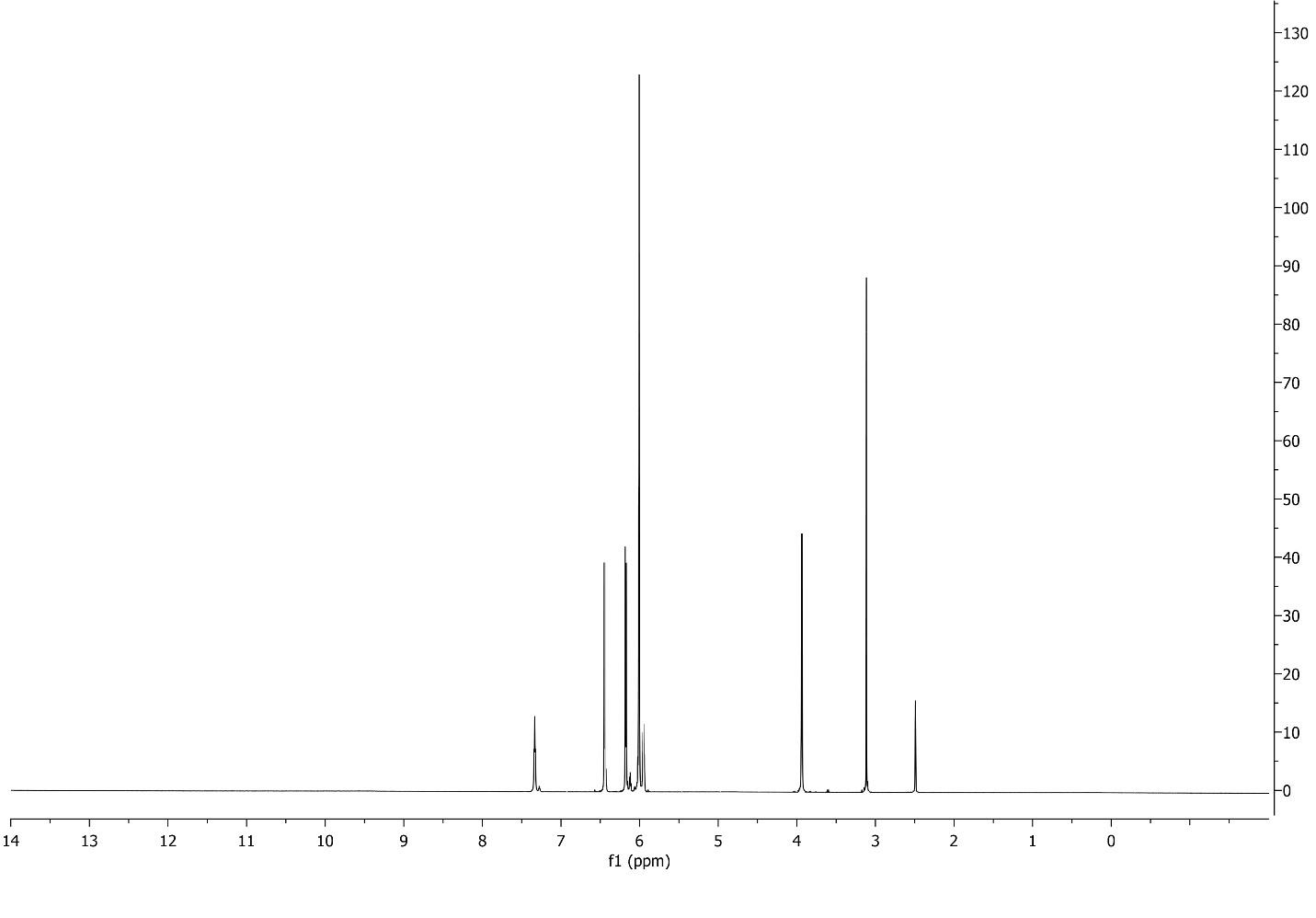
**

[**Benzamide**](javascript:) **(9a)**

FTIR (KBr, cm^-1^): 3361, 3161, 1654, 1572, 1447, 1392, 1176, 629 cm^-1^. ^1^H NMR (500 MHz, DMSO): δ H (ppm)= 8.01(2H, s, NH_2_), 7.89(1H, m, Ar-H), 7.49(2H, m, Ar-H), 7.38(2H, m, Ar-H)


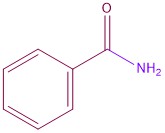

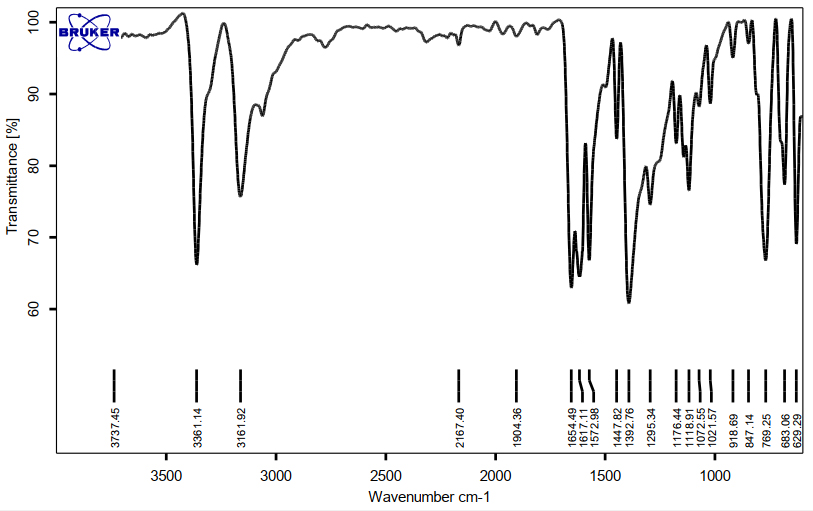


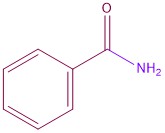

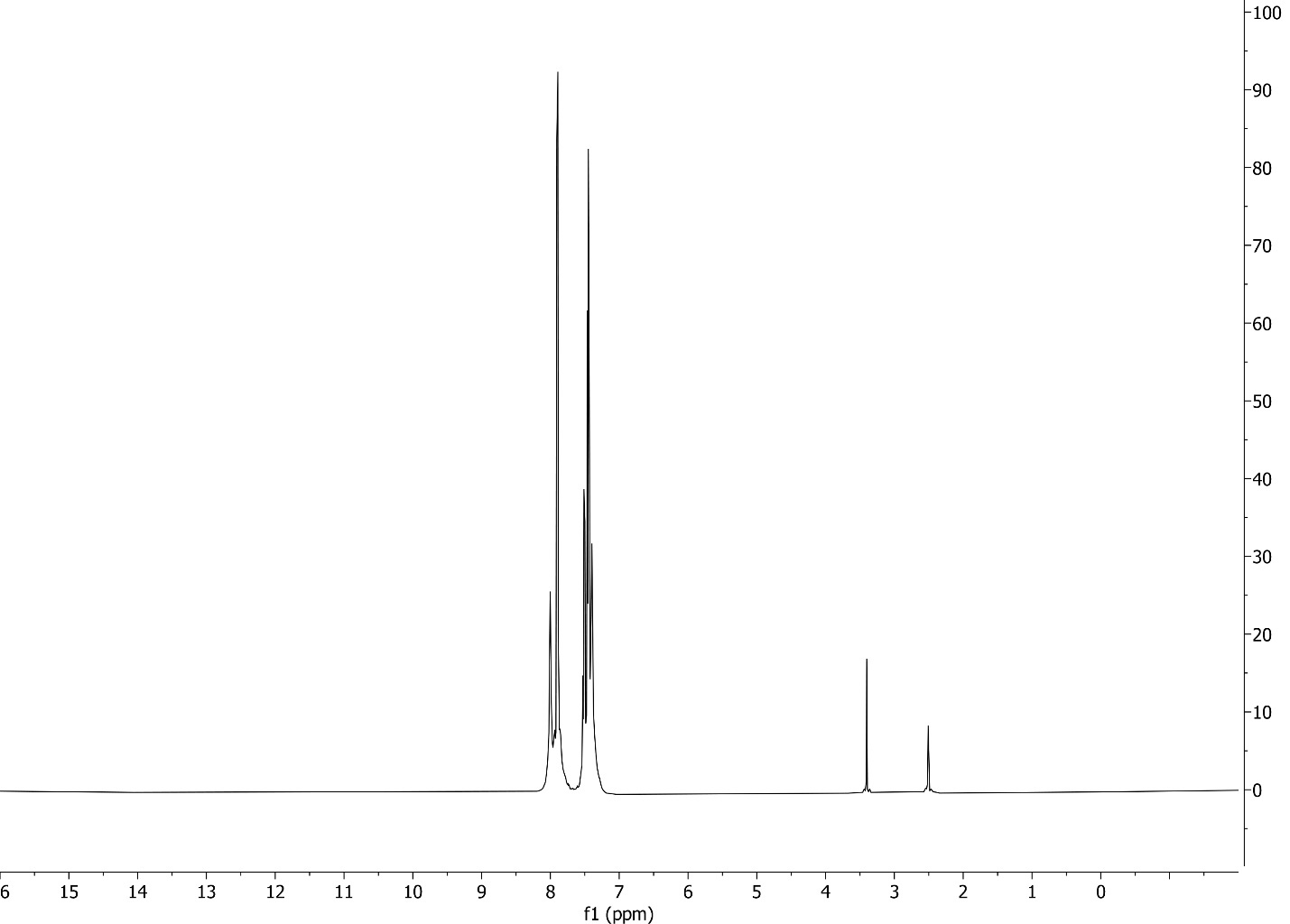


**References**

1. M Eslami, M G Dekamin, L Motlagh, et al. MCM-41 mesoporous silica: a highly efficient and recoverable catalyst for rapid synthesis of α-aminonitriles and imines. *Green Chem. Lett. Rev.* **11**, 36-46 (2018).

2. S Ebrahimi, S Saiadi, S Dakhilpour, et al. N-Acyl-N-(4-chlorophenyl)-4-nitrobenzenesulfonamides: highly selective and efficient reagents for acylation of amines in water. *Z. Naturforsch. B* **71**, 95-104 (2016).

3. K L Manasa, Y Tangella, N H Krishna, et al. A metal-free approach for the synthesis of amides/esters with pyridinium salts of phenacyl bromides via oxidative C–C bond cleavage. *Beilstein J. Org. Chem.* **15**, 1864-1871 (2019).

4. E L Howard, N Guzzardi, V G Tsanova, et al. Highly Efficient Copper‐Catalyzed Amidation of Benzylic Hydrocarbons Under Neutral Conditions. *Eur. J. Org. Chem.* **2018**, 794-797 (2018).

5. K P Patel, E M Gayakwad, and G S Shankarling. Graphene oxide: a convenient metal-free carbocatalyst for facilitating amidation of esters with amines. *New J. Chem.* **44**, 2661-2668 (2020).

6. T K Achar and P Mal. Radical-Induced Metal and Solvent-Free Cross-Coupling Using TBAI–TBHP: Oxidative Amidation of Aldehydes and Alcohols with N-Chloramines via C–H Activation. *J. Org. Chem.* **80**, 666-672 (2015).

7. H Veisi, B Maleki, M Hamelian, et al. Chemoselective hydration of nitriles to amides using hydrated ionic liquid (IL) tetrabutylammonium hydroxide (TBAH) as a green catalyst. *RSC Adv.* **5**, 6365-6371 (2015).

8. A S Hamed and E M Ali. Cu (II)–metformin immobilized on graphene oxide: an efficient and recyclable catalyst for the Beckmann rearrangement. *Res. Chem. Intermed.* **46**, 701-714 (2020).

9. J Chen, Y Xia, and S Lee. Transamidation for the synthesis of primary amides at room temperature. *Org. Lett.* **22**, 3504-3508 (2020).
